# Do the classification of areas and distance matter to the assessment results of achieving the treatment targets among type 2 diabetes patients?

**DOI:** 10.1186/s12942-015-0020-x

**Published:** 2015-09-30

**Authors:** Maija Toivakka, Tiina Laatikainen, Timo Kumpula, Markku Tykkyläinen

**Affiliations:** Department of Geographical and Historical Studies, University of Eastern Finland, P.O. Box 111, 80101 Joensuu, Finland; Institute of Public Health and Clinical Nutrition, University of Eastern Finland, Kuopio, Finland; Hospital District of North Karelia, Joensuu, Finland; Department of Chronic Disease Prevention, National Institute for Health and Welfare (THL), Helsinki, Finland

**Keywords:** Area classifications, Accessibility, Rural health, Urban health, Care outcomes, Type 2 diabetes

## Abstract

**Background:**

Type 2 diabetes is a major health concern all over the world. The prevention of diabetes is important but so is well-balanced diabetes care. Diabetes care can be influenced by individual and neighborhood socio-economic factors and geographical accessibility to health care services. The aim of the study is to find out whether two different area classifications of urban and rural areas give different area-level results of achieving the targets of control and treatment among type 2 diabetes patients exemplified by a Finnish region. The study exploits geo-referenced patient data from a regional primary health care patient database combined with postal code area-level socio-economic variables, digital road data and two grid based classifications of areas: an urban–rural dichotomy and a classification with seven area types.

**Methods:**

The achievement of control and treatment targets were assessed using the patient’s individual laboratory data among 9606 type 2 diabetes patients. It was assessed whether hemoglobin A1c (HbA1c) was controlled and whether the recommended level of HbA1c was achieved in patients by different area classes and as a function of distance. Chi square test and logistic regression analysis were used for testing.

**Results:**

The study reveals that area-level inequalities exist in the care of type 2 diabetes in a detailed 7-class area classification but if the simple dichotomy of urban and rural is applied differences vanish. The patient’s gender and age, area-level education and the area class they belonged to were associated with achievements of control and treatment targets. Longer distance to health care services was not a barrier to good achievements of control or treatment targets.

**Conclusions:**

A more detailed grid-based area classification is better for showing spatial differences in the care of type 2 diabetes patients. Inequalities exist but it would be misleading to state that the differences are simply due to urban or rural location or due to distance. From a planning point of view findings suggest that detailed geo-coded patient information could be utilized more in resourcing and targeting the health care services to find the area-level needs of care and to improve the cost-efficient allocation of resources.

## Background

### Introduction

Type 2 diabetes continues to be a major health burden globally [1]. The changes in lifestyle and in particular the increasing rates of obesity are affecting the increase of diabetes prevalence across the world [1–3]. The prevention of diabetes is important but so is well-balanced diabetes care. Good management of type 2 diabetes improves the quality of life of the patients, reduces complications among patients [4], decreases the risk of comorbidities [5] and reduces the economic burden [6, 7].

Socio-economic inequalities in diabetes care do exist [8]. Achievement of the treatments targets in the diabetes care are affected by individual [9, 10] and neighborhood [11–13] socio-economic status (SES). It is commonly believed that poor geographical accessibility to health care services may lead to delayed care and underuse of health care and this is believed to be the case especially among residents living in rural areas [14]. However, it should be kept in mind that rural health and health in general are interrelated with broader social, economic, political, cultural, historical [15, 16] and spatial structures.

In Finland, primary care is available to all residents and is delivered mainly in public health care centers by general practitioners (GPs). Most of the population lives reasonably close to the nearest health service provider, but in rural areas there are some long distances. In some areas, the availability of public transport is inadequate. However, some of the chronic disease patients are entitled to reimbursements for transportation to be able to attend the regular check-ups.

The aim of the study is to find out whether two different area classifications give significantly different area-level results of achieving the targets of control and treatment among type 2 diabetes patients exemplified by a Finnish region of North Karelia, equivalent in area to New Jersey or 7/10 of Belgium. The focus is to reveal and compare the possible spatial health care divergences by using 2-class (less detailed) and 7-class (detailed) grid based classifications of urban and rural areas. The first hypothesis is that the 7-class classification is better for showing differences in urban and rural areas in the care of type 2 diabetes patients. The second hypothesis claims that the longer the distance to the health center is the more it deteriorates the achievement of control and treatment targets. The study exploits individual geo-referenced type 2 diabetes patient record data from a regional primary health care patient database combined with postal code area-level socio-economic variables, digital road data and grid based classifications of areas.

### Classifications of urban and rural areas

The absence of a generally accepted definition of urban and rural area types makes it difficult to examine spatial health and health care inequalities in a valid way and in particular to compare the results between different countries [17, 18]. This might be one reason for varying results on health and health care inequalities in and between urban and rural areas. It has been suggested that these inequalities should be examined across different settlement types and should not just rely on an urban–rural dichotomy [16, 19, 20].

Often the definitions of urban and rural are based on population density and distance to certain functions such as services [21, 22]. Definitions are usually developed for a certain purpose and generalization can lead to lack of explanatory variation [22]. Traditionally, countries provide the classifications of urban and rural areas based on different indicators, and usually these areas are consistent with the administrative borders such as counties, municipalities, census blocks or census tracts.

However, grid based classifications also exist. In England and Wales urban and rural areas (RUC: Rural–Urban Definition for Small Area Geographies) have been classified for policy purposes by using 1 hectare grid cells [23]. The Organization for Economic Cooperation and Development (OECD) and the European Commission have developed a grid based harmonized definition of cities in Europe which improves cross-country analysis of cities [24]. Grid based and comparable on-task tailored classifications absorb more variation than conventional classifications and thus could be more useful in health related studies.

Helminen et al. [25] have developed a grid based 7-class classification of urban and rural areas for Finland in 2014. Before that, the multiclass classifications of urban and rural areas for various policy purposes were based on municipal borders. Old classification methods became problematic and outdated as many municipalities merged in 2009–2012 creating commuter belts where both urban and rural characteristics could be identified. The new classification procedure is well documented and could be produced for other countries as well.

**Fig. 1:**
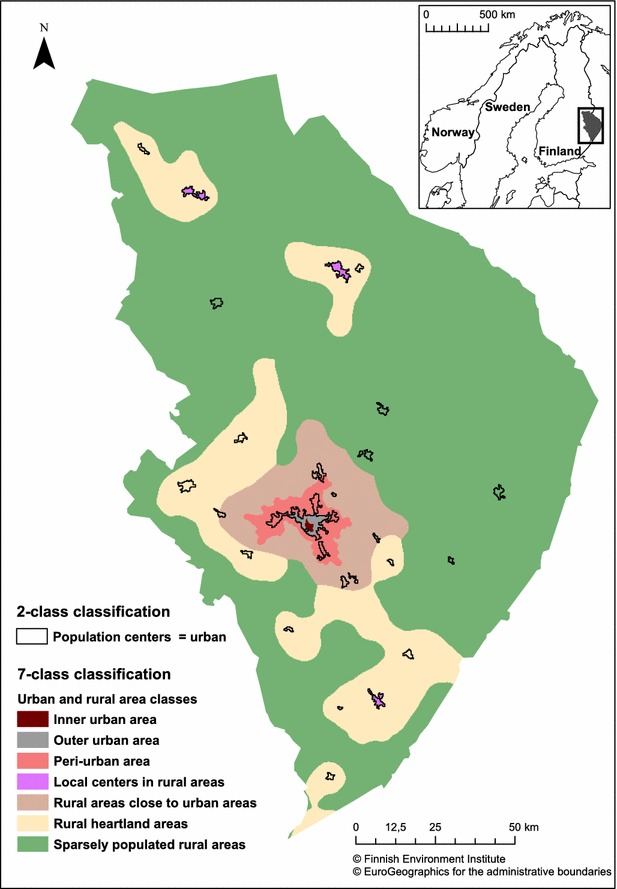
The study region of North Karelia, Finland. The area classifications used in the analyses: the 2-class classification of population centers versus rural areas and the 7-class classification of urban and rural area classes

The 2014 Finnish area classification divides urban areas into three (inner, outer, peri-urban) classes and rural areas into four (local centers in rural areas, rural areas close to urban areas, rural heartland areas and sparsely populated rural areas) classes [26]. It depicts settlement structures focusing on population density, relative location, land use and economic structures. This classification system uses geospatial data represented by a 250 × 250 m grid of cells. Data on population, labor, commuting, buildings, roads and land use have been used. Based on the data, variables describing the amount, density, efficiency, accessibility, intensity, versatility and orientation of the areas have been calculated. Each cell is classified into one of the seven classes according to the defined criteria. All seven area classes are found in the study region described later (Fig. 1).

The Finnish Environment Institute maintains a classification on population centers (known also as statistical locality), which is provided by Statistics Finland. All clusters of buildings with at least 200 inhabitants are defined as population centers [27]. The definition utilizes the building and population data of Statistics Finland’s 250 × 250 m grid data. The definition takes into account the population size, number of buildings and their floor area. The distance between buildings included in population centers is 200 m at maximum with certain exceptions. This categorization was also used in this study to include a simple urban–rural dichotomy (Fig. 1). The patients living in population centers reside in urban areas and the patients living outside the population centers reside in rural areas. The study region of North Karelia is more rural as 70.3 % of the population lived in population centers compared with the Finnish total urban population of 83.7 % in 2012 [28].

### Accessibility of health care services

The accessibility of health care services is affected by the locations of both the health care provider and the patient. According to Penchansky and Thomas [29] accessibility (distance, transportation, travel time and cost) is one of the five dimensions of access among availability (the supply of services), accommodation (hours of operation, waiting times), affordability (price of services) and acceptability (clients’ satisfaction). The poorer the accessibility is the larger the disadvantage is made up by the friction of distance. Further on, accessibility can mean either the potential or revealed accessibility [30–32]. Potential accessibility consists of estimated values often based on surrogate variables, whereas revealed accessibility means the actual use of health care services, thus the health care service utilization [30]. Much of the research is focused on the methodology of measuring potential accessibility [31, 33–37] but less on revealed accessibility and its effects on the outcomes of care.

Transportation options, transportation costs and distance to a health care provider differ depending on the domicile of each patient. Poorer accessibility to health care services is believed to lead to poorer health outcomes [14]. Commonly it is thought that utilization decreases as distance increases. However, the effects of distance vary depending on the health service under consideration [30, 32].

Although distance may influence health care utilization, it is not a barrier to chronic care [38], and patients will travel longer distances for check-ups or for chronic conditions [39]. Mixed evidence is found in the care of diabetes. Driving distance has not been associated with care outcomes within urban settings in Canada [40], and differences in care outcomes have not been found between rural and urban patients in Australia and the USA [41, 42]. In some cases driving distance has been associated with care outcomes in rural areas in the USA [43, 44]. These diabetes related studies used very different definitions of urban and rural or did not define them at all.

## Methods

### Study region and data

The region of North Karelia (13 municipalities, 165,800 inhabitants, population density of 9.3/km^2^) in Eastern Finland is characterized by a regional center (75,000 inhabitants) providing both primary and specialized health care services and there are 24 health care centers in the region. Every patient belongs to a service area of a certain health care center based on the postal code area in which they live.

At the end of the year 2012 approximately 6 % (n = 10,204) of the population in North Karelia had a diagnosed type 2 diabetes. All patient documents have been filed to the regional patient database and individual level data (on e.g. the place of domicile, gender, age, laboratory analyses) was retrieved from this database for every patient who had type 2 diabetes diagnosis (ICD-10 code E11). The data acquisition from the care provider, approved by the ethics committee of the North Savo Hospital District, is described in detail elsewhere [13]. Individual level socio-economic information was minimal, whereupon the postal code area-level data of a patient’s domicile were utilized for describing the socio-economic environment of each patient to see in what kinds of neighborhoods they live. The socio-economic characteristics (Table 1) of the postal code areas were retrieved from the Statistics Finland’s database [45].

**Table 1:** Patients’ mean age and area-level characteristics in different area classes

Area classes	Patients’ mean age	Mean age by area	Educated (%) by areaª	Unemployed (%) by area	Median income (thousands/€) by area
Inner urban area	71.7	43.6	76.6	5.9	16.854
Outer urban area	65.7	39.1	76.3	8.2	18.398
Peri-urban area	64.9	36.0	79.3	5.0	22.740
Local centers in rural areas	69.9	48.2	60.6	7.5	15.817
Rural areas close to urban areas	67.8	40.7	70.8	6.3	18.299
Rural heartland areas	68.2	47.0	60.5	6.9	15.265
Sparsely populated rural areas	67.4	48.4	56.4	7.2	14.936
Population center = urban	68.7	44.3	67.1	7.0	16.941
Outside population center = rural	66.1	45.9	61.6	7.0	15.861

ªAt least high school graduate or vocational training

From the original patient group 94.1 % (n = 9606) were geocoded by address-matching in ArcGIS 10.2.1 software by using Digiroad. The Finnish Transport Agency maintains the national road and street database, Digiroad, which contains precise and accurate data on the location of all roads and streets and address information in Finland. The road distance from a patient’s home to the health care center was calculated using origin-destination cost matrix analysis provided by the software. The travel distance of each patient by road was used because patient record data does not provide information about how the patients usually travel (by car, walking, by public transport). Therefore neither travel time, the way of moving nor the real costs were available.

### Achievement of the control and treatment targets

Finnish Current Care Guidelines form the basis of the treatment and management of diseases and risk factors in health care [46]. Guidelines for diabetes recommends that the hemoglobin A1c (HbA1c) level should be lower than 7.0 % (53 mmol/mol) and that it should be measured every 3–6 months in diabetes patients [47]. HbA1c provides long-term blood sugar levels and it is a good indicator for the good quality of care. It is widely used to measure the outcomes of care [9–13, 48].

In this study, the achievement of the control and treatment targets were assessed by the realization of a control measurement and the achievement of the recommended HbA1c level. As the HbA1c should be measured regularly, it was assessed whether HbA1c was measured during the years 2011–2012 among the type 2 diabetes patients. Only the latest measurement was used. The clinical outcomes of care were categorized as good [HbA1c <7 % (<53 mmol/mol)] and poor [HbA1c ≥7 % (≥53 mmol/mol)] HbA1c levels among those patients whose HbA1c was measured. Only patients, who had at least 3 months between their diabetes diagnosis and their last HbA1c measurement were included in the analyses to guarantee an appropriate period for treatment effect.

### Statistical analyses

First, a Chi square test (χ^2^) of independence was used to compare differences in the achievement of the control and treatment targets between different area classes. At very first, it was tested whether HbA1c was measured equally often in the seven area classes. Next, we tested the differences of the measurement frequencies of patients living in and outside a population center. A similar test of independence was performed to investigate whether HbA1c was under the recommended level of less than 7 % in patients in the seven area classes and in patients living in or outside a population center.

Second, logistic regression analysis was used to test which variables affect the probability that HbA1c is measured and the probability that HbA1c is less than 7 % (dependent variables). Both the patient’s individual characteristics (patient’s age, gender and road distance from a patient’s home to the health care center) and the patient’s neighborhood characteristics (the percentage of educated people, the percentage of the unemployed and the median income) were set as independent variables. The 7-class classifications of urban and rural areas were used as independent variables so that sparsely populated rural areas was the reference category. Additionally, the dichotomous variable of living in a population center was used as an independent variable.

## Results

The average road distance from a patient’s home to the health care center was 2.1 km in population centers and 14.9 km outside them. The longest distance was 92 km. The majority (approx. 70 %) of patients were living within a 5-km radius from the health care center. Table 2 presents the results of the dependence of the achievement of the control and treatments target by the seven area classes and Table 3 presents the results by the simple dichotomous variable of urban and rural.

**Table 2:** Realization of the control measurement and achieving the recommended level of HbA1c by 7-class area classification

7-class classification of areas	Numbers of patients and their areal percentage distribution	Proportions of HbA1c measured patients^a^ to the diagnosed (%)	Proportions of HbA1c <7 % patients^a^ to the measured (%)	Patients’ mean driving distances and the ranges^b^ in km
Inner urban area	849 (8.8 %)	82.8	74.8	2.0 (0–4.0)
Outer urban area	1433 (14.9 %)	80.5	75.6	2.1 (0–9.5)
Peri-urban area	644 (6.7 %)	85.6	74.8	5.0 (0.1–27.1)
Local centers in rural areas	1414 (14.7 %)	79.9	69.2	1.8 (0–5.7)
Rural areas close to urban areas	725 (7.5 %)	84.6	71.8	7.8 (0–27.9)
Rural heartland areas	2376 (24.7 %)	84.9	73.1	6.0 (0–36.0)
Sparsely populated rural areas	2165 (22.5 %)	83.5	66.7	12.1 (0–91.8)
Total	9606 (100 %)			5.9 (0–91.8)

^a^χ^2^ p value <0.05

^b^Minimum and maximum values in brackets

**Table 3:** Realization of the control measurement and achieving the recommended level of HbA1c by 2-class area classification

2-class classification of areas	Numbers of patients and their areal percentage distribution	Proportions of HbA1c measured patients to the diagnosed (%)	Proportions of HbA1c <7 % patients to the measured (%)	Patients’ mean driving distances and the ranges^a^ in km
Population center = urban	6754 (70.3 %)	83.0	72.0	2.1 (0–22.5)
Outside population center = rural	2852 (29.7 %)	83.0	70.7	14.9 (1–91.8)
Total	9606 (100 %)			5.9 (0–91.8)

^a^Minimum and maximum values in brackets

The best control measurement rates were found in peri-urban areas, rural heartland areas and rural areas close to urban areas (Fig. 1). In all of these classes approximately 85 % of the patients had gone through the HbA1c measurement. The weakest situation was in local centers in rural areas where HbA1c was measured in 79.9 % of the patients. The best results for HbA1c level lower than 7.0 %, were found in outer and inner urban areas and peri-urban areas. The worse outcomes of care were found again in local centers in rural areas and especially in sparsely populated rural areas. The differences between the seven area classes related to the achieved treatment targets were statistically significant. However, there were no statistically significant differences in existence of the control measurement or achieving the recommended level of HbA1c when the dichotomous variable of urban and rural was under investigation (Table 3). The control measurement results (83 %) were the same for both urban and rural patients but urban patients achieved the recommended level of HbA1c a little more often, but as mentioned, the result was not statistically significant.

Table 4 presents which variables affect the probability that HbA1c is measured and that HbA1c is less than 7 % tested by the logistic regression models. The variables that remained statistically significant at the level of p value below 0.05 are included in the table. The probability of HbA1c measurements increased with ageing. The level of education in the neighborhood also increased the probability of attendance at HbA1c screenings. Compared with patients in inner urban areas, outer urban areas and local centers in rural areas, patients in sparsely populated rural areas had their HbA1c measured more often. Female gender and younger age increased the probability of achieving the recommended HbA1c level of 7 %. Surprisingly, the model suggests that when the road distance from a patient’s home to the health care center increases it is more probable that HbA1c is less than 7 %. Additionally, when sparsely populated rural areas are compared with other area classes, all the other areas perform better in achieving the recommended level of HbA1c. The dichotomous variable of population centers did not remain in the model.

**Table 4:** Effect of patient characteristics, area-level factors and area classes on achieved treatment targets

Variable	Is HbA1c measured? (0 = no, 1 = yes)	HbA1c level (0 = 7 % and over, 1 = less 7 %)
Gender (0 = male, 1 = female)		1.22 (1.10–1.35)
Age	1.02 (1.02–1.03)	0.99 (0.99–1.00)
Educated (%)	1.02 (1.01–1.04)	
Unemployed (%)		
Median income (thousands/€)		
Distance (km)		1.01 (1.00–1.02)
Inner urban area	0.57 (0.39–0.83)	1.63 (1.32–2.03)
Outer urban area	0.56 (0.40–0.79)	1.64 (1.36–1.97)
Peri-urban area		1.53 (1.22–1.91)
Local centers in rural areas	0.67 (0.56–0.81)	1.23 (1.02–1.46)
Rural areas close to urban areas		1.33 (1.08–1.65)
Rural heartland areas		1.42 (1.23–1.65)
Sparsely populated rural areas	Reference category	Reference category
Pop. center (0 = outside, 1 = inside)		
R^2^	0.022	0.014

The logistic regression models revealing the effects of patient characteristics, neighborhood characteristics, area classes or the dichotomy of urban and rural on the HbA1c control measurement and the achievement of the recommended HbA1c level. The odds ratios (OR) with confidence intervals (CI) of the variables that remained statistically significant (p < 0.05) in the models are presented in the table

The findings from logistic regression models confirm the findings from Tables 2 and 3 that a more refined area classification reveals spatial differences in the achievement of the control and treatment targets. If the patient group is merely divided into the two categories of urban and rural, no differences are found in the achievement of the control or treatment targets. If the broader, more detailed classification of urban and rural is used, differences between specific area types are observed.

## Discussion

The aim of the study was to find out whether the area classifications significantly influence the area-level results of achieving the targets of control and treatment among type 2 diabetes patients. The results have been statistically tested to understand the risk in the interpretation of results in the research area. As most phenomena are geographically contingent [49], we do not aim at making generalizations about the likely transferability of findings to other regions although statistically significant results indicate that similar differences are possible to exist elsewhere.

Our first research task was to clarify whether differences in the achievement of the control and treatment targets among type 2 diabetes patients exist in different area classes. We applied 2-class and 7-class grid based classifications of urban and rural areas. When the simple dichotomy of urban and rural was used, no differences were found in the achievement targets assessed by the realization of the control measurement and the achievement of the recommended HbA1c level. The detailed classification with seven different area classes revealed statistically significant spatial differences in the achievement of control and treatment targets. These results strongly indicate that it is more informative to apply a more refined area classification than a simple urban–rural dichotomy, as has also been suggested earlier [16, 19, 20]. The comparison of achievement of control and treatment targets in diabetes care within a detailed area classification can help to identify areas at risk in a finer scale. Different results from 2-class and 7-class classifications of urban and rural areas indicate that the classification methods and classification principles chosen can easily affect the results and conclusions. Classifications can even be contradictory. For instance, several population centers in the 2-class classification of urban areas belong to rural heartland areas or sparsely populated rural areas in the 7-class classification (Fig. 1). The different choices of areal units (whether it is based on administrative borders, grids or something else) affect the results, which should be kept in mind especially when comparing studies and results between countries.

The analyses for the testing of the second hypothesis revealed that differences in the existence of control measurement between urban and rural areas were not due to the remote location of the rural patients as the road distance from a patient’s home to the health care center was not a significant factor in explaining the control measurement rate. For the achievement of recommended HbA1c level, the model suggested that when the distance increases it is more probable that the recommended HbA1c level is achieved. This clearly states that the distance is not a barrier to good control or to achieve treatment targets.

Even though rural patients have to travel from longer distances to health care services than their urban counterparts, the distances are not that hindering in the care of type 2 diabetes in the study region. In Australia, for example, the distances can be much longer, producing a bigger barrier to the accessibility of health care [41]. In case of a need for control measurement the visits to the health care services can be planned beforehand and combined with other errands run in local centers. Moreover, the National Health Insurance scheme, which is part of the Finnish social security system, provides the reimbursement of health care related travel costs and accepts travel by taxi if a patient is unable to use a less expensive mode of transport for health reasons or if public transport is not available [50].

The analyses revealed some additional findings and issues. Firstly, regardless of the distance, inequalities in diabetes care exist, and these are partly due to a patient’s individual characteristics such as gender and age. Also urban and rural neighborhoods seem to matter which reflect individual characteristics and status to a high degree. However, these individual and area-level variables explain only a small part of the variation. Clearly, local differences exist but it would be misleading to state that these differences are simply due to the urban or rural location. Smith et al. in their review [51] indicate that rurality per se does not necessarily lead to rural–urban disparities, but for example it may exacerbate the effects of socio-economic disadvantages. This can be observed for example when economic growth takes place predominantly in cities leading to selective migration of the healthier. Inequalities by area types in health care mainly stem from differences in their socio-economic and demographic characteristics [16, 19, 21], originated from their socio-economic legacy and selective migration [52–54]. This can be seen to be the case in our study region as well. One of the limitations of this study was that we were not able to analyze socio-economic characteristics and the ways of life (values, norms, nutrition etc.) on an individual level as these particulars are not available in patient records.

Secondly, the service structure of the health care system and the processes of care are important factors to achieve efficient care results. Even though clinical guidelines are a national standard and largely implemented, the performance in health care is hardly ever homogenous. Performance gaps evolve when society develops. The study indicates that combining information from different databases is cost-effective in comparison with surveys and it can be useful in planning, resourcing and targeting primary health care services. In this study, we were neither able to assess service structures or different processes of care nor to get individual socio-economic characteristics of the patients. These factors might explain one part of the differences found in the achievement of control and treatment targets in diabetes care. Also patient’s motivation to his/hers own care can play an important role in achieving the treatment targets. If such data could be inserted into patient record databases, future research could examine these factors cost-efficiently.

## Conclusions

In conclusion, individual and area-level differences exist in the achievement of control and treatment targets of care of chronic conditions such as type 2 diabetes even in an area with a relatively homogenous public primary health care system. Geographical accessibility seems not to be a deteriorating factor in the care of type 2 diabetes. The patient’s gender and age, area-level education as a surrogate and the area class they belonged to were associated with achievement of control and treatment targets, and thus such information could be utilized much more in planning, resourcing and targeting the health care services. However, these factors explained only a small part of the variation. More information on the impact of processes and resources in health care and individual level characteristics are needed to obtain a comprehensive picture of factors predicting variation in the outcomes of care, but even so area-level information seems to be suggestive, at least for small-area health care planning.
